# The potential of predictive and prognostic breast MRI (P2-bMRI)

**DOI:** 10.1186/s41747-022-00291-z

**Published:** 2022-08-22

**Authors:** Matthias Dietzel, Rubina Manuela Trimboli, Moreno Zanardo, Rüdiger Schultz-Wendtland, Michael Uder, Paola Clauser, Francesco Sardanelli, Pascal A. T. Baltzer

**Affiliations:** 1 Department of Radiology, University Hospital Erlangen, Maximiliansplatz 3, 91054 Erlangen, Germany; 2 Humanitas Clinical and Research Center — IRCCS, Via Manzoni 56, Rozzano, Milan, Italy; 3 Department of Biomedical Sciences for Health, Università degli Studi di Milano, Via Mangiagalli 31, 20133 Milan, Italy; 4 Division of Molecular and Gender Imaging, Department of Biomedical Imaging and Image-Guided Therapy, Medical University of Vienna, Waehringer-Guertel, 18-20 Vienna, Austria; 5 Unit of Radiology, IRCCS Policlinico San Donato, Via Morandi 30, San Donato Milanese, 20097 Milan, Italy

**Keywords:** Biomarkers, Breast neoplasms, Magnetic resonance imaging, Precision medicine, Prognosis

## Abstract

Magnetic resonance imaging (MRI) is an important part of breast cancer diagnosis and multimodal workup. It provides unsurpassed soft tissue contrast to analyse the underlying pathophysiology, and it is adopted for a variety of clinical indications. Predictive and prognostic breast MRI (P2-bMRI) is an emerging application next to these indications. The general objective of P2-bMRI is to provide predictive and/or prognostic biomarkers in order to support personalisation of breast cancer treatment. We believe P2-bMRI has a great clinical potential, thanks to the *in vivo* examination of the whole tumour and of the surrounding tissue, establishing a link between pathophysiology and response to therapy (prediction) as well as patient outcome (prognostication). The tools used for P2-bMRI cover a wide spectrum: standard and advanced multiparametric pulse sequences; structured reporting criteria (for instance BI-RADS descriptors); artificial intelligence methods, including machine learning (with emphasis on radiomics data analysis); and deep learning that have shown compelling potential for this purpose. P2-bMRI reuses the imaging data of examinations performed in the current practice. Accordingly, P2-bMRI could optimise clinical workflow, enabling cost savings and ultimately improving personalisation of treatment. This review introduces the concept of P2-bMRI, focusing on the clinical application of P2-bMRI by using semantic criteria.

## Key points

- Magnetic resonance imaging (MRI) is an essential imaging modality for the assessment of breast diseases; it investigates the entire tumour volume *in vivo* as well as the surrounding tissue and the whole breast(s) providing imaging biomarkers for both prediction and prognostication.
- Predictive breast MRI may establish the link between imaging information and therapeutic decision-making.
- Prognostic breast MRI may enable us to foresee the patient outcome.
- Predictive and prognostic breast MRI (P2-bMRI) reuses already performed MRI examinations and does not require additional invasive tissue sampling or potentially expensive analytic procedures.
- P2-bMRI promises great benefits to clinical workflow, allowing cost savings and personalisation of treatment.

## Background

Magnetic resonance imaging (MRI) represents an important part of multimodal breast imaging [1–6]. Based on three-dimensional multiparametric imaging, it provides high soft tissue contrast enabling functional insights into the pathophysiology of breast disease [2, 6]. Such qualities translate into its unsurpassed sensitivity and negative predictive value [7, 8].

Multiple indications to perform MRI in clinical practice are established and may be summarised by few key questions of breast imaging: “Is there a lesion?” (detection in both screening and diagnostic scenarios); “Is the lesion malignant?” (characterisation and problem-solving), “Where is the lesion located? Are there are other suspicious ipsilateral or contralateral lesions? How much is the disease extended in relation with the breast volume?” (preoperative locoregional staging), and “Is the tumour responding to neoadjuvant therapy?” (treatment monitoring) [3, 4, 9–11]. These clinical indications are mostly accepted worldwide. The only exception concerns the role of breast MRI in preoperative locoregional staging. The final results of a large multinational investigation (the MIPA study) recently provided important real-world data on this matter [10, 12], but this indication remains a matter for debate.

Predictive and prognostic breast MRI (P2-bMRI) is an emerging application next to these indications. Generally, P2-bMRI may be approached as an umbrella term summarising tools aimed at one general objective. Such general objective of P2-bMRI is to provide predictive and/or prognostic MRI biomarkers. Such imaging biomarkers may ultimately support the personalisation of breast cancer treatment [13]. P2-bMRI applies a wide spectrum of tools to achieve this general objective: standard and advanced multiparametric pulse sequences, structured reporting criteria (for instance BI-RADS descriptors), artificial intelligence methods, including machine learning (with emphasis on radiomics data analysis), and deep learning that have shown compelling potential for this purpose [14–19].

P2-bMRI is different to traditional biomarkers; it does typically not require additional patient examinations but recycles imaging data already available from routine breast MRI [20, 21]. Accordingly, P2-bMRI may transform breast MRI into a one-stop shop examination, hence providing both diagnostic and predictive/prognostic information. Already now, MRI is routinely performed in many state-of-the-art breast imaging units, for example for preoperative staging. In this case, data required for P2-bMRI are already available in an great number of patients [3, 9]. Whereas alternative biomarkers typically rely on invasive tissue sampling and may require potentially expensive analytical procedures, this is not the case for P2-bMRI [20–22]. Thus, P2-bMRI holds great promises related to patient workflow, treatment personalisation, and cost-effectiveness of breast cancer treatment.

This narrative review introduces the concept of P2-bMRI and presents its potential advantages. Tools available for P2-MRI are discussed. Hereby, special focus is set on the clinical application of P2-bMRI by using semantic criteria.

## The role of P2-bMRI in the perspective of P4 medicine

Personalised, predictive, preventive, and participatory (P4) medicine is a key concept for state-of-the-art oncology [23]. In breast cancer care, P4 medicine aims to tailor therapy to the individual patient and the specific tumour biology. In order to translate P4 medicine into clinical practice, new diagnostic methods and refinement of existing tools are required [23, 24].

P2-bMRI can be used to provide imaging biomarker supporting personalisation of breast cancer diagnosis and treatment, *i.e.*, screening strategies based on patient-based data and therapies based on specific tumour- and patient-based data. Therefore, P2-bMRI may become an important driver for the translation of P4 medicine into clinical practice. In the following, we summarise the concept of precision medicine and discuss how P2-bMRI will help to bridge critical research gaps in this field [24].

### Precision medicine

Precision medicine aims to adopt therapy based on specific characteristics of the individual patient, including disease susceptibility, biology, and prognosis as well as response to treatment [13, 24–26]. Molecular subtyping is a classic example how specific biological characteristics of breast cancer aid personalisation of patient treatment in current clinical practice [13]. Hereby, molecular subtyping provides decision support on whether and what systemic therapy should be appropriate, such as endocrine therapy in luminal cancers, targeted therapy in human epidermal growth factor receptor 2 (HER2)-positive cancers, or immune therapy in triple negative cancers [13, 27]. Precision medicine may be further improved by genetic microarrays. It has been demonstrated that genetic microarrays can distinguish patients who ultimately benefit from cytotoxic treatment from those women where chemotherapy may safely be omitted [20, 28]. Regardless of these advances, personalisation of therapy is still at a relatively early stage of development, judged to be a critical research topic [24]. Although steps forward in this direction were done in the last decades, the contribution of noninvasive techniques such as MRI is a highly interesting option to be considered [29–31].

### Prognostic and predictive biomarkers and their relevance for precision medicine

Biomarkers are critical elements for the development of precision medicine [24, 32]. Eccles et al. [24] called for the development of “imaging biomarkers” in a systematic gap analysis on most urgent breast cancer research topics. Authors expect that “validation of multimodality imaging biomarkers” will provide a better understanding of biological breast cancer behaviours, hereby supporting the personalisation of treatment [24].

Per definition, any specific “characteristic that is measured as an indicator of normal biological processes, pathogenic processes, or responses to an exposure or intervention, including therapeutic interventions” may be regarded as a potential biomarker [32, 33]. Two types of biomarkers are of special interest for the concept of precision medicine: *prognostic* and *predictive* biomarkers. The former provides information about overall disease outcome. They may be used to identify patients who actually may benefit from certain types of treatments. Whereas prognostic biomarkers do not provide information about which individuals are likely to benefit from a specific therapy, this may be achieved by predictive biomarkers. Accordingly, predictive biomarkers may support clinicians in selecting the most appropriate type of treatment for the individual patient [34–36]. While this differentiation (prognostic *versus *predictive biomarkers) is relevant, we should consider that there is an obvious interplay between prognostication and prediction. With reference to the P4 medicine [21], we should consider that the second P (“predictive”) includes both prognostication outcome prediction.

### Biomarkers from breast MRI

Application of P2-bMRI as a source of prognostic and predictive biomarkers can aid personalisation of treatment. This ultimately may bridge critical research gaps in the successful treatment of breast cancer [24]. In comparison with traditional biomarkers, such as histopathological (type/grading) and molecular and genetic examinations (receptor status, multigene arrays), MRI biomarkers offer specific advantages due to the intrinsic characteristics of the method, as specified below.

1. P2-bMRI investigates the whole tumour *in vivo.* P2-bMRI hereby potentially reducing the risk of sampling errors [37, 38]. Moreover, also, the surrounding tissue (*e.g.*, background parenchymal enhancement [BPE] and peritumoural environment, especially oedema, as described below) and the whole breast(s) can be potentially considered. In contrast, conventional biomarkers may rely on samples taken from specific selected tumour regions.
2. P2-bMRI does not require invasive tissue sampling and allows to visualise the tumour *in vivo*. It is commonly well-tolerated by patients and has no absolute contraindications except those related to the presence of unsafe ferromagnetic implanted and/or electronic medical devices.
3. P2-bMRI may transform breast MRI in a one-stop shop solution providing both diagnostic and predictive/prognostic information. While the indication for preoperative MRI is still the subject of debate, it is already regularly performed in clinical practice [9, 10, 12]. In these patients, the data required for P2-bMRI are readily available, and costly additional investigations are not required. Accordingly, P2-bMRI promises significant cost savings for treatment personalisation. In contrast, alternative tumour profiling methods are known cost drivers, so limiting their broader application as recently argued to Bhargava et al. [22].
4. P2-bMRI provides intrinsic advantages to the clinical workflow as all data could be available in real time.
5. Predictive/prognostic data from P2-bMRI can be complementary to conventional biomarkers. So, P2-bMRI may fine tune the prognostic assessment of patients, which have been initially assessed by conventional biomarkers [39].

### Triaging patients by P2-bMRI

Application as a gatekeeper is a promising use case of P2-bMRI. Here, the method will serve as a triage tool to select patients for more advanced procedures of precision medicine such as genetic testing [22, 38]. Triaging breast cancer patients for genetic testing have been advocated by Bhargava et al. [22]. Authors argue that genetic tests are costly, not generally available, and changes in treatment affect only a subgroup of patients [22]. They have developed triage tools to safely forego molecular testing based on standard histological examinations [22]. Validation studies demonstrated that triaging can obviate molecular testing in the majority of patients without compromising oncologic safety. At the same time, cost savings of US $3,000 are achieved for every skipped molecular test [22]. Accordingly, Bhargava et al. [22] expect an enormous healthcare value of triage tools in the era of precision and P4-medicine.

The correlation of MRI data with molecular profiling and genetic tests has been independently verified by many authors [40–42]. Hence, P2-bMRI may be adopted as a triage tool similar to the concept of Bhargava et al. [22]. As previously described, P2-bMRI provides results in real time without the need of additional invasive and analytic procedures. Therefore, we expect a potential of P2-bMRI as a triage tool in precision medicine, such as a valuable help for selecting patients for genetic testing.

## Technical requirements for P2-bMRI

**Fig. 1:**
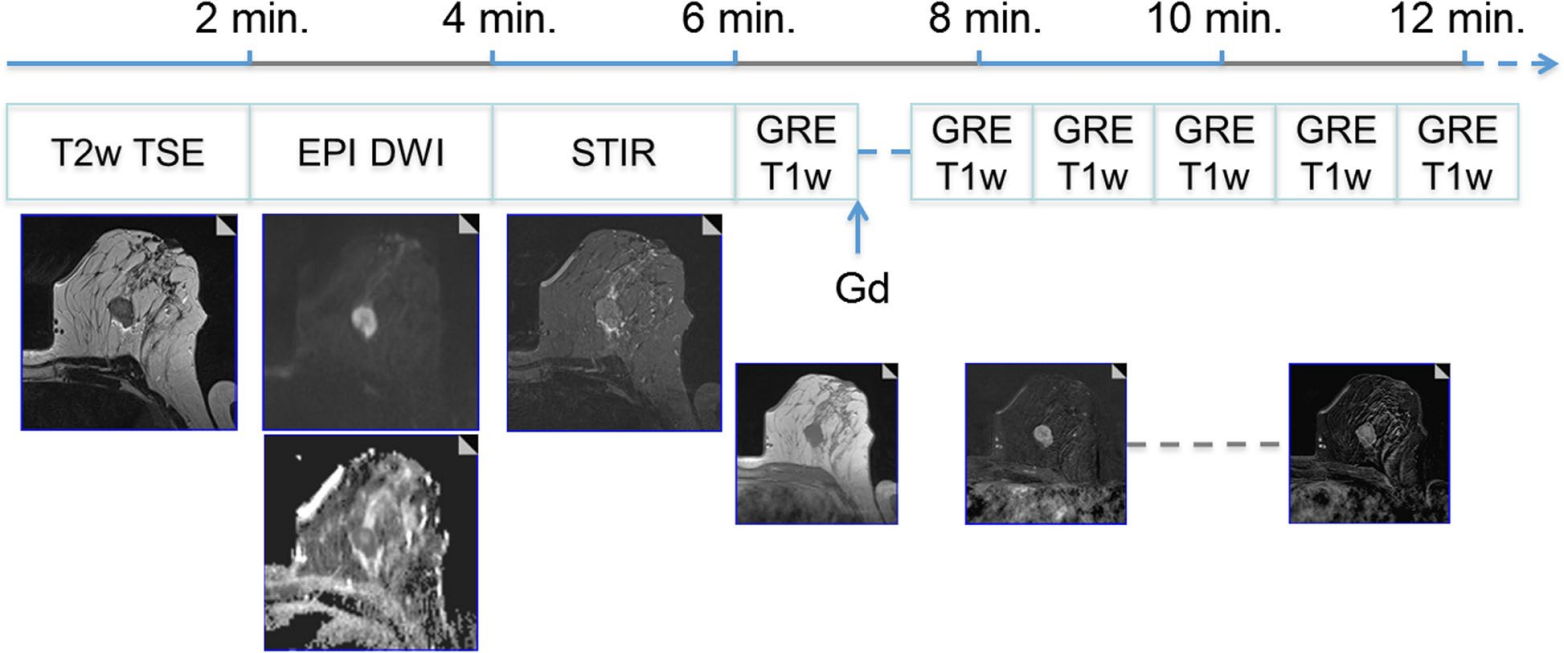
A 15-min clinical protocol for breast magnetic resonance imaging (MRI). All predictive/prognostic breast MRI information demonstrated in the next figures can be derived from a one-stop shop clinical protocol as shown in this figure. The protocol starts with an unenhanced T2-weighted turbo spin-echo sequence (T2w TSE). Diffusion-weighted imaging (DWI) and short-tau inversion recovery (STIR) are optional but highly recommend. On T2-weighted images, a mass lesion is diagnosed, with perifocal oedema. Next, contrast-enhanced dynamic scanning is performed using a T1-weighed gradient-echo (GRE) sequence before/after the intravenous administration of 0.1 mmol/kg of a Gd-based contrast agent. There is evidence of washout, perifocal oedema, and central necrosis (rim sign). The last two descriptors are imaging biomarkers associated with increased probability of high-grade and nodal-positive invasive cancers. Washout is a strong predictor of poor outcome and is associated with a higher likelihood of metachronous metastasis (see also Figs. 5 and 6). Example taken from ref [2], with permission (Dietzel et al. *Insights Imaging* 2018)

Technical requirements for an up-to-date P2-bMRI are the same as for any diagnostic breast MRI [2, 4]. An overview is given in Fig. 1. In short, a state-of-the-art full protocol breast MRI protocol should include a T2-weighted sequence, a diffusion-weighted sequence, and a dynamic T1-weighted sequence (*i.e.*, before/after intravenous application of a gadolinium-based contrast agent) [52, 53]. Particular attention should be given to the quality of diffusion-weighted images, possibly taking into consideration the recommendations provided by the European Society of Breast Imaging [5]. Specialised sequences such as spectroscopy and fast sequences for pharmacokinetic analysis are promising to improve future performance of P2-bMRI [65–68] but are not performed outside specific research projects.

## Data analysis of P2-bMRI

Just like in diagnostic MRI itself, the spectrum of tools available for P2-bMRI is broad as well; it ranges from semantic criteria to advanced post-processing techniques, such as artificial intelligence, including radiomics data analysis [14–19]. Generally artificial intelligence may address a wide range of clinical use cases including predictive/prognostic tasks [14, 15]. The status of radiomics and artificial intelligence in breast imaging extends beyond the aim of this article and has been reviewed previously [14, 15, 19, 69]. There is no doubt that these methods offer a great advantage for P2-bMRI [14, 15, 17–19]. At the current stage, however, these methods are reserved for academic institutions and are not yet suitable for widespread clinical use. Published data are still insufficiently validated independently and externally, which is why the generalisability has not yet been proven [16].

## P2-bMRI: semantic criteria

**Fig. 2:**
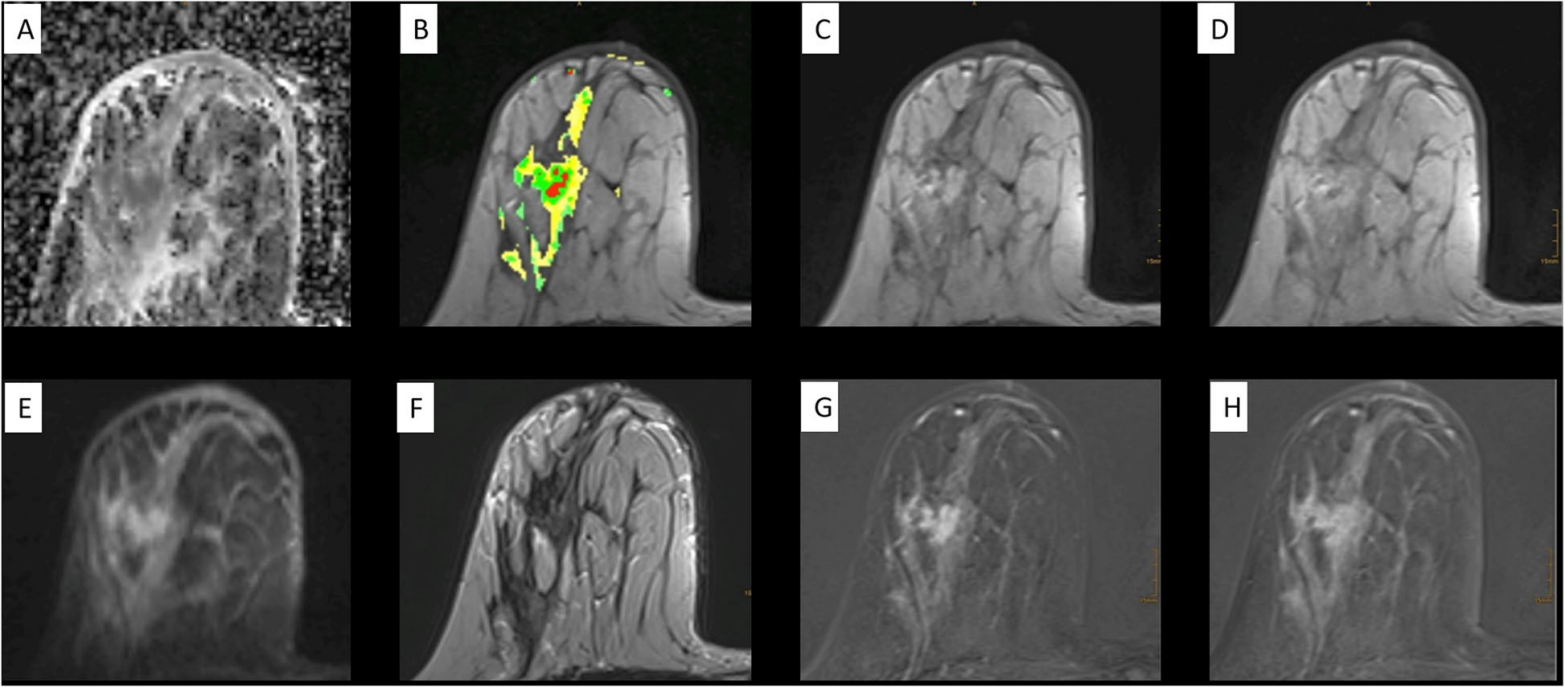
Standardised reading setup for comprehensive diagnostic and predictive/prognostic breast magnetic resonance imaging (MRI) at one-stop shop. A female patient with suspicious amorphous segmental calcifications on the right breast at mammography, breast imaging reporting and data system (BI-RADS) 4 diagnostic category (not shown). MRI was performed also for preoperative staging due to suspicion of extended ductal carcinoma *in situ*. Diagnostic MRI shows an extensive heterogeneous segmental non-mass enhancement predominantly with plateau dynamic pattern. A noncircumscribed mass with washout and heterogeneous internal enhancement (BI-RADS 5 diagnostic category) is located centrally within the non-mass lesions. Relevant prognostic findings are here washout, skin thickening, invasion of the nipple, and diffuse ipsilateral oedema (see also Figs. 5 and 6). Semantic criteria correspond to the MRI phenotype of an aggressive invasive breast cancer. P2-MRI results were confirmed by postoperative pathological examination (invasive cancer NOS, G3, Ki-67+++, triple negative, node positive). Apparent diffusion coefficient map (**A**); unenhanced T1-weighted gradient echo with colour overlap of the dynamic curve on a pixel-by-pixel basis (green/yellow/red = persistent/plateau/washout) (**B**); first (**C**) and last (**D**) contrast-enhanced T1 GRE; diffusion-weighted imaging obtained with *b* = 800 s/mm^2^ (**E**); T2-weighted turbo spin-echo (**F**); first (**G**) and last (**H**) contrast-enhanced subtractions. Diffusion-weighted imaging findings are highlighted in Fig. 7

**Fig. 3:**
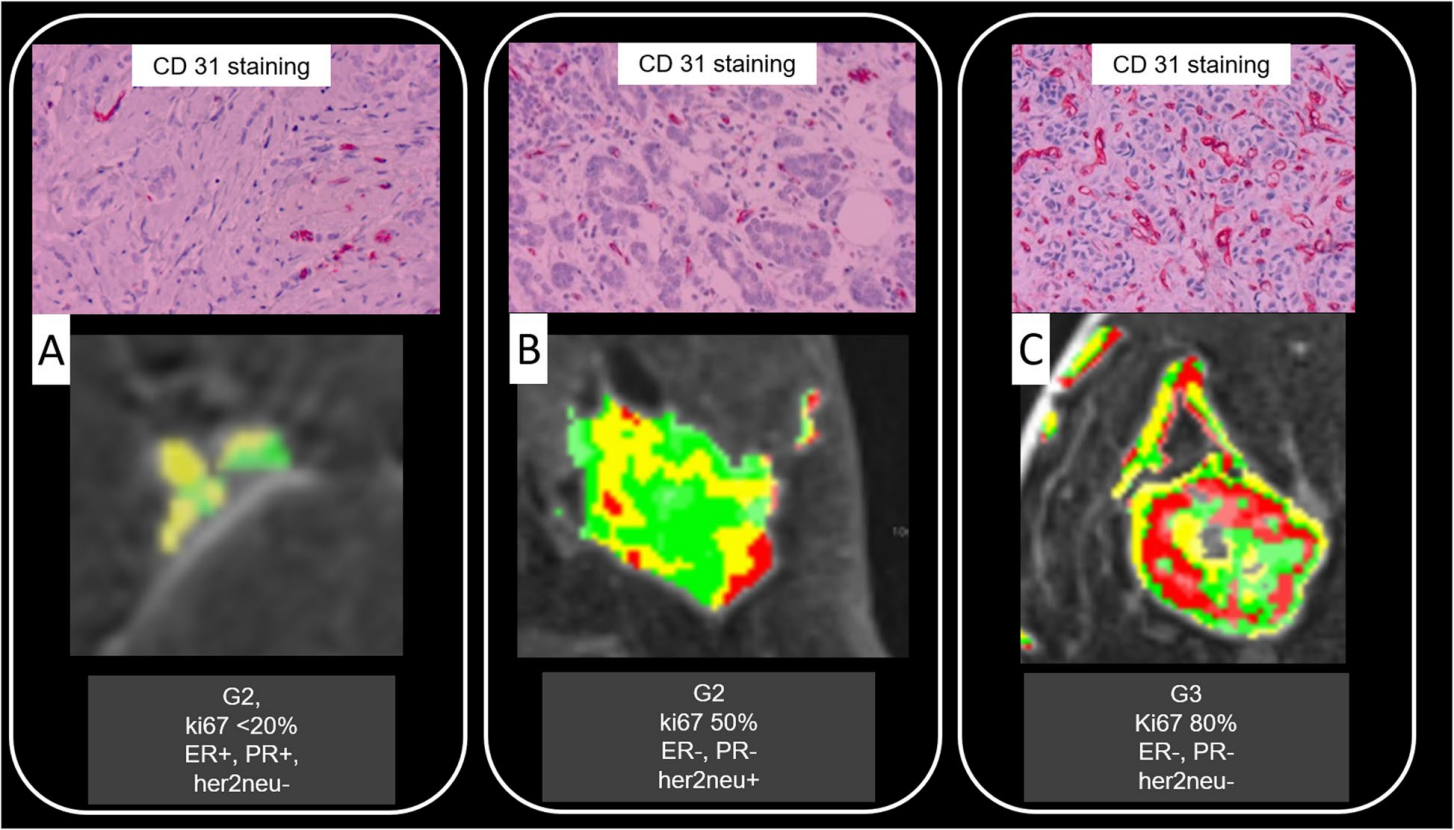
Benefit of vascular analysis for predictive/prognostic breast magnetic resonance imaging. In **A**, **B**, and **C**, the enhancement patterns of three different breast cancers are colour coded on a pixel-by-pixel basis (green/yellow/red = persistent/plateau/washout). The maps reveal tumour heterogeneity, progressively increasing from **A** to **C** (no washout pixels in **A**, few of them in **B**, and many in **C**). Findings correspond to an increasing risk profile which was verified upon pathological and molecular analysis. Here, increasing vascularisation (CD 31 staining top row), cellular proliferation (Ki-67), and aggressiveness (grading) was demonstrated, and a less favourable receptor profile was evident from **A** to **C**. *ER* Oestrogen, *her2neu* Human epidermal growth factor receptor 2, *PR* Progesterone

**Fig. 4:**
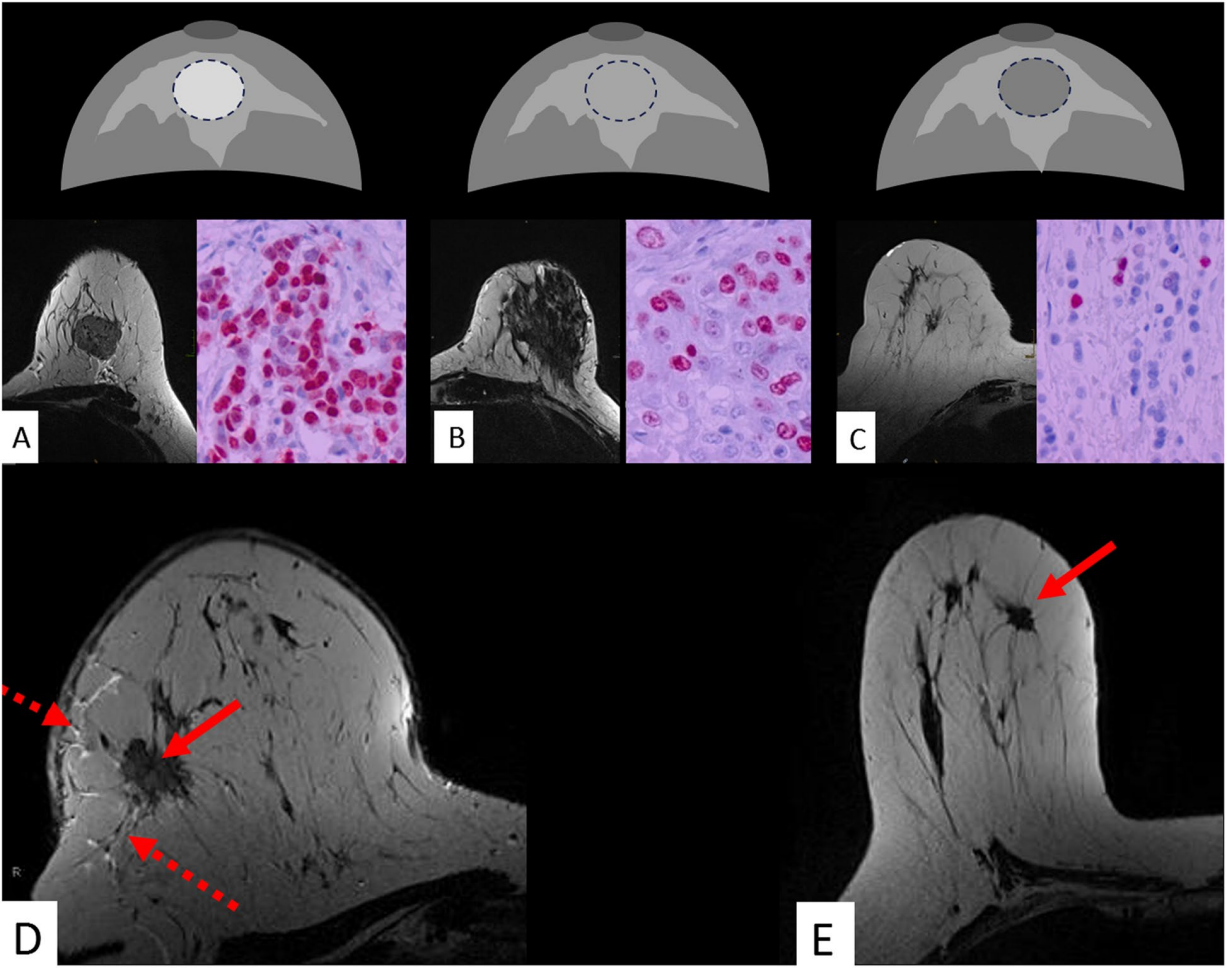
Potential role of T2-weighed images: the lesion signal as a possible surrogate of water content. Signal intensity on T2-weighted images may reflect water content and warrants further scientific research. Compared to the surrounding parenchyma, a lesion is classified as hyperintense (**A**), isointense (**B**), or hypointense (**C**). In **D**, a mass lesion is identified on T2-weighted images (red arrow). T2-weighted signal intensity is assessed in comparison with the surrounding parenchyma. In **D**, just like the adjacent Cooper ligaments, the parenchyma displays less signal than the tumour. Correspondingly, the tumour is classified as “hyperintense”. Findings suggest the presence of an aggressive breast carcinoma phenotype. Predictive/prognostic findings were verified by immune histology revealing high-grade cancer with negative steroid receptors and elevated Ki-67 suggesting high cellular proliferation. Note perifocal and subcutaneous oedema (dotted arrows). In contrast, **E** displays a hypointense less aggressive carcinoma (red arrow, G2, positive steroid receptors, only Ki-67+)

**Fig. 5:**
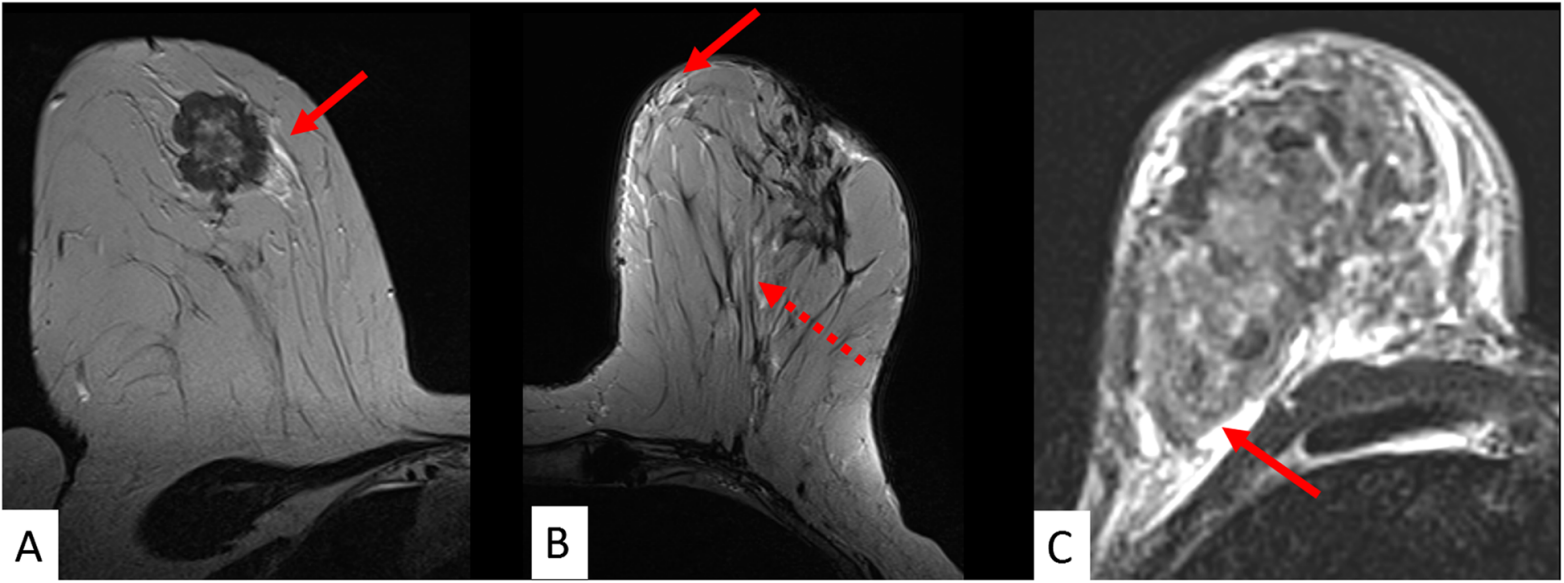
The pivotal role of T2-weighed images: oedema. Any asymmetric ipsilateral T2-weighted signal increase not due to the tumour itself, cysts or artefacts is referred to as “oedema” in **A**, **B**, and **C**. Different oedema patterns are distinguished such as perifocal (**A**), full red arrow), subcutaneous (**B**, full red arrow), prepectoral (**C**, full red arrow), and diffuse (**B**, dotted red arrow). Oedema is considered among the best evaluated predictive/prognostic criteria and was associated with high grade and nodal-positive cancers as well as disease recurrence (see also Table 1)

**Fig. 6:**
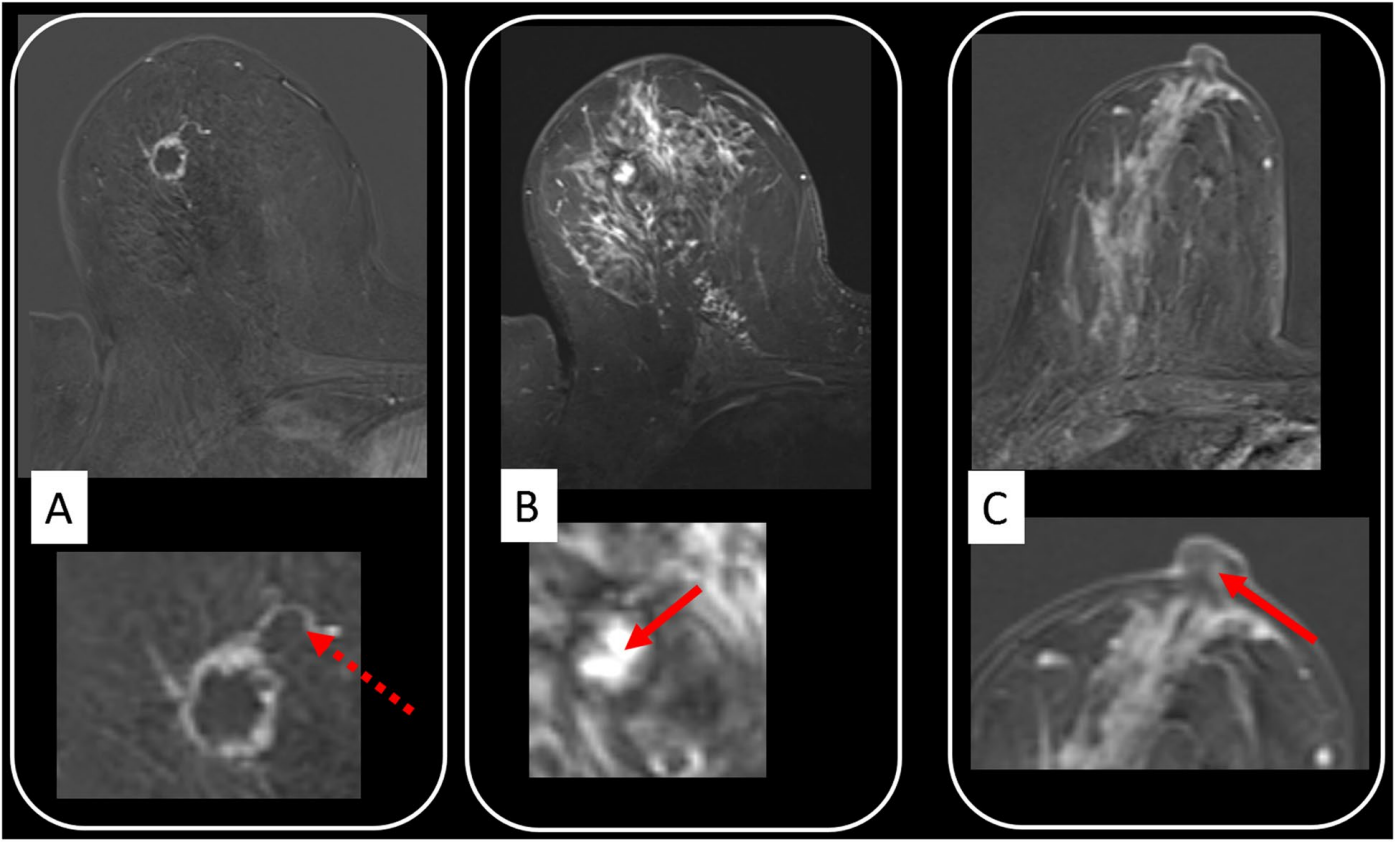
Selected semantic parameters with known biological correlates. They can be readily implemented into clinical practice. Rim enhancement (**A**) was among the first morphologic parameters reported in the literature and reflects an aggressive cancers phenotype. This is also suggested by the adjacent vessel sign (**A**, magnification: dotted red arrow) [50]. Rim enhancement is thought to reflect central hypovascularity due to connective tissue, fibrosis, and/or necrosis. Necrosis sign (**B**) specifically depicts central colliquative (liquid) necrosis (**B**, magnification: full red arrow), characterised by a high signal intensity on T2-weighted images within the centre of the tumour. Invasion of the cancer into the nipple areolar complex is related to poor outcome. The semantic criterion described as “destruction of nipple line” (**C**) is best depicted on DCE images (**C**, magnification: red arrow). Further details including diagnostic performance of semantic parameters are provided in Table 1

**Fig. 7:**
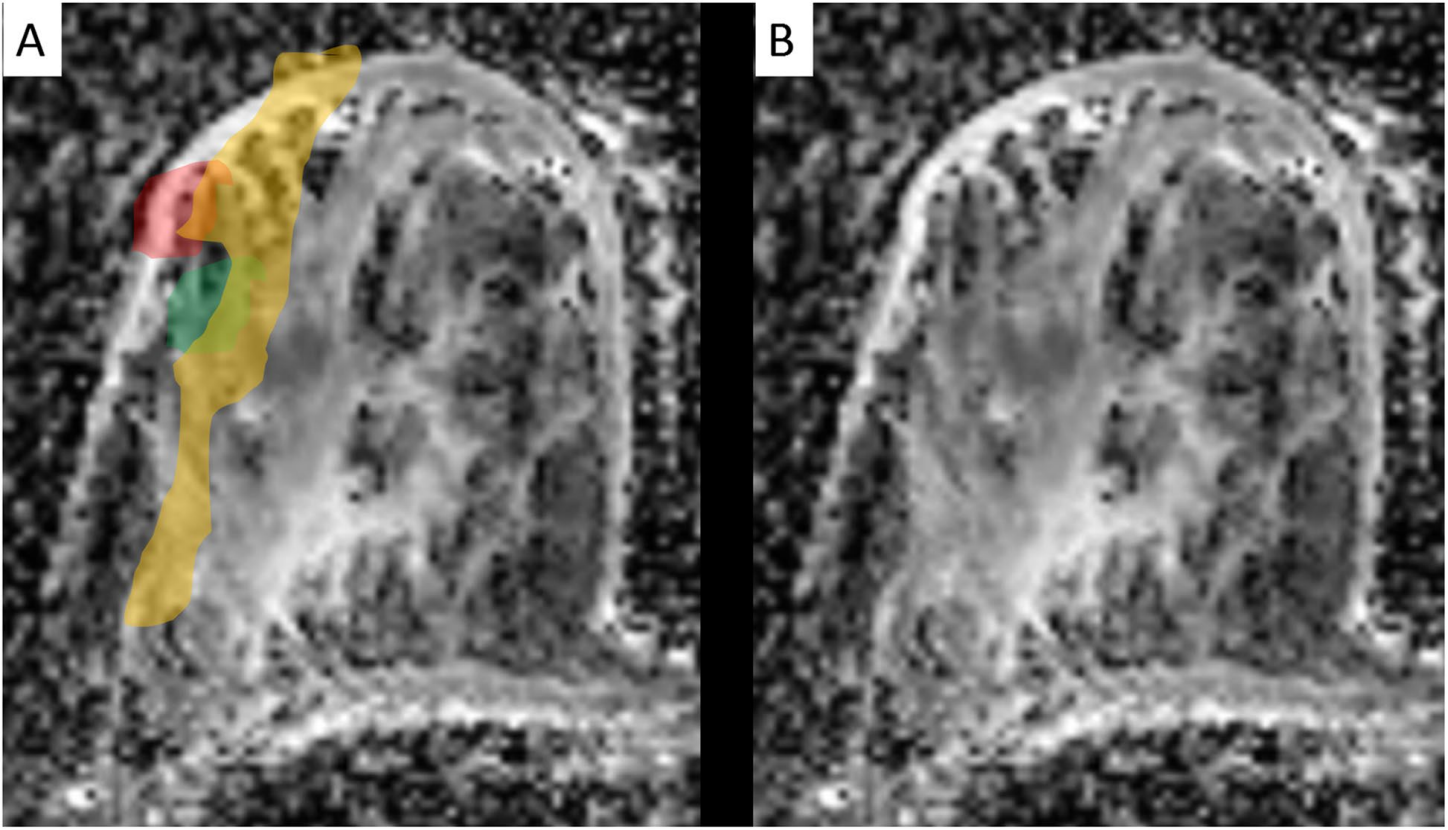
Apparent diffusion coefficient (ADC) mapping (detailed analysis of the DWI already shown in Fig. 1). ADC values are extracted using quantitative region-of-interest-based measurements by standardised methods. ADC is given with (**A**) and without (**B**) colour overlap. ADC was applied to distinguish ductal carcinoma *in situ* (DCIS, orange) from invasive carcinoma (red). Note the presence of a benign lesion (a fibroadenoma), also correctly characterised by the ADC map (green), adjacent to the DCIS

In contrast, semantic criteria are an integral part of routine breast MRI diagnostics [43]. This enables us to apply P2-bMRI in a large number of patients already today. In the following, we give an overview of how to apply semantic P2-bMRI criteria to clinical breast MRI protocols. Figures 1, 2, 3, 4, 5, 6, and 7 and Table 1 summarise key concepts of this approach.

**Table 1:** Semantic criteria of predictive/prognostic breast MRI

Criterion	Acquisition	Assessment	Pathophysiological correlate	Predictive/prognostic value		References
				Comment	Statistics	
Amount of fibroglandular tissue	DCE, T2WI	Visual (American College of Radiology classes from *a* to *d*) or automated	Fibroglandular tissue, stromal matrix, dense connective tissue, collagen, elastin, lobules, and ducts	One of the strongest independent biomarkers of breast cancer incidence. The prognostic value is proven only for mammography. Similar effect for MRI is expected	Relative risk (%: amount of fibroglandular tissue on mammograms) for the four classes: a) 1.79 (< 25%) b) 2.11 (25–50%) c) 2.92 (50–75%) d) 4.64 (> 75%)	[43–45]
Background of parenchymal enhancement	DCE	Visual (1st dynamic scan) or automated	Tissue perfusion due to hormonal stimulation and proliferative activity	For high-risk women, positive correlation with BC incidence. No association among women with average risk	High risk and at least mild background parenchymal enhancement: odds ratio 2.1	[46–49]
Adjacent vessel sign	DCE	Visual	Hypervascularisation Neoangiogenesis	Presence of adjacent vessel sign indicates invasive cancer. It is less common in DCIS	Invasive cancer or DCIS? DOR 2.7; specificity 72.6%	[50, 51]
Destruction of nipple line	DCE	Visual (Fig. 8)	Invasion of the nipple-areola complex	“Destruction of nipple line” is associated with nodal-positive breast cancer	Is this cancer likely to show lymph node metastasis? DOR 2.5; specificity 88.5%	[52, 53]
Oedema	T2WI	Visual (Fig. 6) Perifocal Prepectoral Subcutaneous Diffuse	Changes in the tumour habitat Cytokine effects Vessel permeability Lymphovascular dissemination Pitfalls: double check with patient history; renal, cardiac origin (possible bilateral diffuse oedema of non-neoplastic origin); treatment-related (surgery, radiation therapy)	Presence of “diffuse unilateral oedema” is a strong predictor of nodal-positive and high-grade breast cancer	Is this cancer likely to show lymph node metastasis? Specificity 94.9%; DOR2.6 Is this cancer high grade (G3) or not (G1 or G2)? Specificity 95.5%; DOR 2.4	[39, 52, 54–57]
				Perifocal oedema is also an independent predictor of disease recurrence	Is this patient likely to develop disease recurrence? Hazard ratio 2.48	
Lesion type	DCE	Visual according to breast imaging reporting and Data system descriptors: mass, non-mass, or “mixed” (mass and non-mass)	Unknown	Cancers revealing both mass and non-mass enhancement (“mixed”) are more often associated with lymphovascular invasion (compared to mass or non-mass)	Is this cancer associated with lymphovascular invasion? DOR 2.4; specificity 82.7%	[58]
				Cancers revealing mass-like enhancement are more likely to be HER2-positive (compared to non-mass and mixed)	Is this cancer HER2-positive? DOR 2.7; specificity 85.7%	
				Non-mass invasive ductal cancers are more likely to be low grade (compared to mass and mixed)	Is this invasive ductal cancer low grade (G1) or not (G2 or G3)? DOR 9.3; specificity 85.3%	[59]
Necrosis sign	T2WI	Visual: hypointense lesion with hyperintense centre	Central colliquative (liquid) necrosis	Presence of necrosis sign indicates high-grade invasive cancers	Is this cancer high grade (G3) or not (G1 or G2)? Specificity 94.3%; DOR 3.7	[60]
Skin thickening	Unenhanced T1WI	Visual	Subcutaneous tumour spread, inflammatory tumour	Presence of skin thickening indicates high-grade invasive cancers. It is less common in G1 and G2 cancers Presence of skin thickening is also a strong predictor of lymph node metastasis	Is this cancer likely to show lymph node metastasis? DOR 5.9; specificity 94.5%	[52, 53]
Rim sign	DCE	Visual	High microvessel density in the peripheral zone of the vital tumour. Connective tissues, fibrosis, and/or necrosis at central part of the tumour centre	Presence of rim sign is associated with an increased risk of lymph node metastasis and high-grade cancer	Is this cancer likely to show lymph node metastasis? DOR 2.7; specificity 57.1% Is this cancer high grade (G3) or not (G1 or G2)? DOR 6.1; specificity 57.5%	[61]
Signal intensity	T2WI	Visual (Fig. 7): compared to unaffected breast gland parenchyma: hypointense, isointense, or hyperintense	Water content of the lesion	Hyperintensity on T2WI is associated with elevated Ki-67 and increased cellular proliferation	Is this cancer likely to show high (Ki-67 ≥ 14%) or low proliferative activity (Ki-67 < 14)? DOR 2.2; specificity 59.8%	[62]
Washout	DCE	Visual, region of interest, or computer-assisted	Hypervascularisation Neoangiogenesis Arteriovenous shunts (anarchic vascularisation)	A high washout rate (> 40%) is associated with an increased risk of metachronous metastasis	Is this patient likely to develop metachronous metastasis? Sensitivity 100%; negative predictive value 100%	[63, 64]

Values reported in the “statistics” column express the probability of a certain outcome (*e.g.*, “nodal metastasis present”), when the given MRI criterion is present (*e.g.*, “washout present”, “mass lesion present”) derive from the referenced literature

*DCE* Dynamic contrast-enhanced study, *DOR* Diagnostic odds ratio, *HER2* Human epidermal growth factor receptor 2, *T1WI* T1-weighted imaging, *T2WI* T2-weighted imaging

### Background parenchymal enhancement

The vascularisation of normal breast parenchyma is assessed by BPE [43]. In high-risk women, BPE has been identified as a prognostic imaging biomarker of breast cancer risk; women showing at least “mild” BPE are associated with significantly greater odds of future breast cancer (odds ratio: 2.1), which may be explained by deficient tissue repair mechanisms in this subgroup of women [46, 47] (Table 1). However, among average-risk women, the level of BPE is not associated with a higher risk of breast cancer [46].

The association of current BPE with breast cancer prognostic factors, such as higher mammographic density, steroid receptor status, and lymphovascular invasion has been reported in the literature [70]. Lim et al. [68] (hazard ratio 3.1) and Choi et al. [69] (postmenopausal; hazard ratio 3.9) independently reported the association of BPE with recurrence-free survival in average-risk patients with [70, 71]. These data emphasise the future potential of BPE as a genuine MRI imaging biomarker in the personalisation of breast cancer care.

### Tumour enhancement: morphology and dynamics

A broad spectrum of semantic criteria is available to characterise breast tumour vascularisation. They may be applied to P2-bMRI as well [43, 50, 63, 72]. Neovascularisation is considered a key step in the process of carcinogenesis [73]. Patterns of neovascularisation can be assessed by microvessel density at traditional pathology examination, and this parameter is regarded a prognostic biomarker of breast cancer by itself [74]. Contrast enhancement is the basis for MRI diagnosis of breast cancer and is thought to reflect tissue vascularisation (Fig. 3) [2, 43]. Accordingly, many authors hypothesised that MRI enhancement patterns correlate with patient outcome and eventually may be used as imaging biomarkers [38, 40, 75].

*Wash out* is a key diagnostic criterion of the delayed enhancement phase but should also be approached as a prognostic biomarker [2, 43, 63]. It has been identified as a powerful tool to rule out the risk of metachronous metastasis (sensitivity and negative predictive value 100%; criterion, washout rate > 40%) [63]. Although these findings have to be validated in clinical trials, results highlight the potential of P2-bMRI parameters to estimate individual patient risk profile (Fig. 3, Table 1).

Breast cancer is a heterogeneous disease [37]. *Volumetric analysis* of MRI enhancement parameters investigate the composition of the entire tumour vasculature and are considered an imaging correlate of breast cancer heterogeneity [38, 76] (Fig. 3). Accordingly, the association of volumetric MRI parameters with histopathology and prognostic factors of breast cancer such as lymph node, hormonal receptor, and HER2 status has been demonstrated [40]. Building upon these results, correlation of volumetric MRI enhancement patterns with surrogate-free measure of patient outcome has been demonstrated in the meantime [38, 76]. For example, P2-bMRI has been shown to predict overall survival of breast cancer patients [76]. In a subsequent study, authors demonstrated that volumetric analysis of MRI enhancement yielded synergistic effects to conventional biomarkers [38]. Findings support the hypothesis that P2-bMRI can be used as add-on tool to further refine risk stratification of established prognostic biomarkers.

*Rim enhancement* is a classic diagnostic pattern of breast MRI [2, 43] (Fig. 6). Its prognostic value was early reported in the literature. Jinguiji et al. [61] investigated the relationship of this semantic MRI criterion with prognostic factors. Authors reported the significant association of rim enhancement with multiple prognostic factors such as lymph node metastasis, blood vessel invasion, steroid receptors, tumour size, and histological grade (G3 *versus* G1 or G2: diagnostic odds ratio 6.1; specificity 57.5%) (Table 1) [61]. Rim enhancement is thought to reflect central hypovascularity due to the presence of connective tissue, fibrosis, and/or necrosis in rapidly growing aggressive cancers [60, 61]. On the other hand, the *necrosis sign* is considered to indicate colliquative (fluid) necrosis, a pattern characterised by high signal intensity inside the cancer on T2-weighted scans [60]. As outlined in Table 1, necrosis sign has been described as one of the most specific semantic MRI criteria of high grade cancers (G3 *versus* G1 or G2 cancers: diagnostic odds ratio 3.7; specificity 94.3% (Table 1) [60].

### Biomarkers from unenhanced breast MRI

*T2-weighted* s*ignal intensity* of a breast cancer is classified as hyper-, iso-, or hypointense compared to the surrounding breast tissue (Fig. 5, Table 1). In P2-bMRI, this semantic criterion serves as a predictor of tumour proliferation. Biologically less active desmoplastic tumours typically exhibit hypointense signal intensity on T2-weighted scans. In contrast, the presence of a T2-weighted hyperintense cancer suggests increased cellular proliferation and elevated Ki-67 expression (diagnostic odds ratio 2.2, specificity 59.8%) [62, 77].

While rim enhancement and signal intensity on T2-weighted scans investigate the gross anatomy of breast cancer, tumour microstructure can be investigated by diffusion-weighted imaging (DWI)*.* DWI patterns are quantified by the apparent diffusion coefficient (ADC), which is typically used as a quantitative biomarker to aid differential diagnosis of suspicious breast lesions [5, 78, 79]. DWI could be used for P2-bMRI as well, and we regard the assessment of tumour invasiveness by ADC mapping as a promising clinical application [80]. Bickel et al. [80] reported higher ADC levels for ductal carcinoma *in situ* (DCIS) compared to invasive cancers. Personalisation of DCIS treatment is based on core biopsy samples, which are known to miss invasive tumour components in a relevant number of patients. Importantly, pure DCIS tumours require a different treatment strategy, which is why delaying correct diagnosis of invasive cancers should be avoided. To solve this dilemma, ADC may be used as a decision support tool [80]. Different to core biopsy, DWI examines the whole tumour, reducing the risk of sampling errors, which leads us to the following use case: if presurgical histology reveals DCIS, but ADC values (as well as patterns of contrast enhancement) are suggestive of invasive cancer, diagnosis of pure DCIS has to be questioned [80]. In this scenario, re-biopsy shall be considered and may avoid delayed diagnosis of invasive cancer as proposed by Bickel et al. [80] (Fig. 7).

### Associated vascular findings

The *adjacent vessel sign* is a finding related to the macrovasculature of breast lesions. According to Dietzel et al. [70], the adjacent vessel sign reflects invasiveness of breast cancer. It indicates the presence of invasive cancer and is rarely seen in DCIS (diagnostic odds ratio 2.7, specificity 72.6%) [50] (Fig. 6, Table 1).

Whole breast vascular maps were investigated by Sardanelli et al. [72, 81] showing the association of an increased unilateral map with the presence of invasive cancers. This association was also shown to allow an increase in specificity using a 3-T magnet [82]. Martincich et al. [63] studied the variations of vascular maps in the context of primary systemic therapy. They showed that before therapy, vascular maps were asymmetrically increased ipsilaterally to the locally advanced breast cancer. After primary systemic therapy, vascular maps significantly changed only in the breast harbouring the cancer, with responders showing significantly more reduce vascular maps than nonresponders [63].

### Associated nonvascular findings: oedema

The local tumour environment is recognised as a key factor in breast cancer development. It may be studied with semantic MRI criteria [83]. Oedema is defined as an associated finding in the BI-RADS lexicon, and it is characterised by T2-weighted signal increase within the local tumour environment [43, 54] (Fig. 5). It can be classified as perifocal, diffuse, subcutaneous, and prepectoral [39, 54–56, 84]. In general, the presence of oedema is indicative of aggressive cancer phenotypes, which is especially true for diffuse and prepectoral patterns [54, 55, 84]. According to Kaiser et al. [85], the latter is typically associated with lymph node metastases, lymphangitic carcinomatosis, and invasion of the chest wall. Subcutaneous oedema is specific of inflammatory breast cancer [84]. Whereas the majority of studies on the prognostic value of oedema used surrogates of patient outcome [54–56, 84], Cheon et al. investigated the impact of perifocal oedema on patient outcome [37]. Authors identified perifocal oedema as an independent biomarker of disease recurrence (hazard ratio 2.48) potentially improving the prognostication of disease recurrence by conventional biomarkers [39]. Meanwhile, the prognostic value of the semantic criterion “oedema” has been verified by numerous authors [86–88]. In particular, advanced techniques such as high-resolution diffusion-weighted imaging and radiomics showed promising potential to analyse peritumoural tissue, and they may further support the clinical impact ofP2-bMRI [89–91].

### Tumour extent

Accurate assessment of anatomical tumour extent is the main rationale for preoperative breast MRI locoregional staging [10, 92]. However, this assessment provides significant prognostic information as well; *tumour size* is a key prognostic factor of breast cancer, and larger tumours are associated with a higher likelihood of worse outcome [92, 93]. Infiltration of breast cancer into associated structures such as the nipple areola complex or into the skin is associated with a poorer patient outcome [92, 94]. Accordingly, Dietzel et al. [52, 53] demonstrated that semantic criteria such as *destruction of nipple line* (diagnostic odds ratio 2.5, specificity 88.5%) or *skin thickening* (diagnostic odds ratio 5.9, specificity 94.5%) are associated with a poor prognostic profile such as that defined by the presence of locoregional lymph node metastases (Fig. 6, Table 1).

Occurrence of locoregional and distant metastasis deteriorates prognosis [93, 95]. Whole body MRI is an established tool to detect breast cancer metastasis, and its performance may be further improved by utilising the technology combining positron emission tomography and MRI [96–98]. While typical preoperative MRI aims to primarily assess ipsilateral tumour extension and the possibility of contralateral breast cancers, state-of-the-art scanner hardware could actually combine dedicated breast MRI examination with whole-body examinations as suggested by Kirchner et al. [98]. Since significant prognostic information can be derived from whole body MRI examinations, they are promising in the context for P2-bMRI as well [93, 95]. Different strategies exist for whole body examinations in breast cancer care. Kirchner et al. [98] proposed a complete whole body positron emission tomography/MRI staging. On the other hand, abbreviated protocols enabling screening for breast cancer metastasis are available as well [99, 100]. Requiring only 90 s of additional examination time, such protocols can be combined with a standard breast MRI within one single examination. Initial clinical data demonstrated promising results regarding both lymph node staging (positive predictive value of 100%, negative predictive value of 94.3%) and distant metastases screening (sensitivity 100%, specificity 98.3%) [99, 100].

### Breast MRI phenotyping

Highly accurate breast MRI diagnosis is not achieved, until multiple parameters are assessed in concert [1, 2]. The same applies to P2-bMRI. Although individual parameters already enable prognostic assessment (Table 1), the relevance of P2-bMRI can be further specified when the lesion is analysed in the concert of multiple parameters [52, 99]. If these patterns correspond to a specific tumour biology, we refer to this feature combination as P2-bMRI phenotype. Accordingly, P2-bMRI phenotypes can provide actionable information, which is why we expect their key role in translating P2-bMRI into clinical practice.

**Fig. 8:**
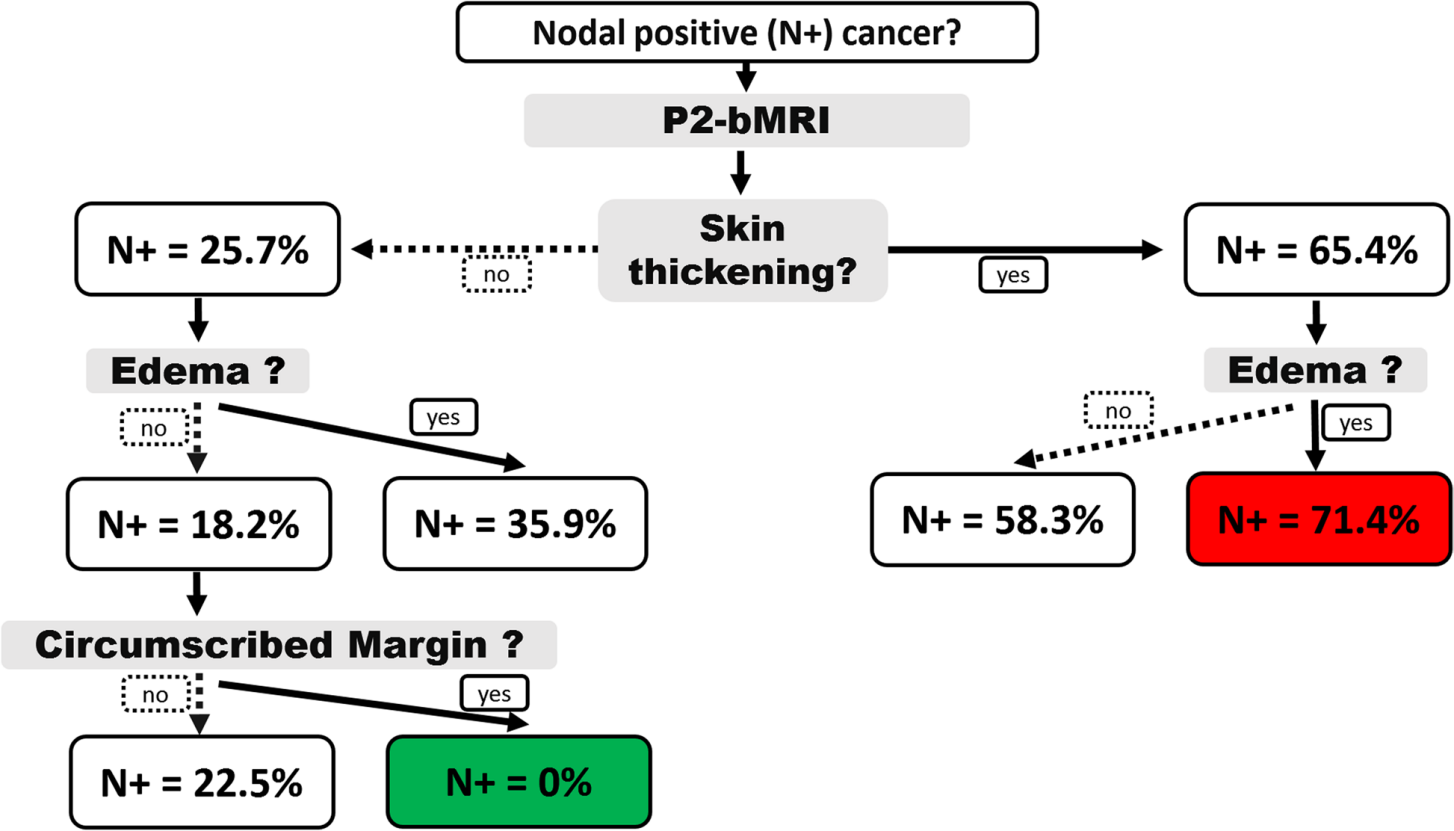
P2-bMRI phenotypes are imaging patterns highly specific of a distinct tumour biology. They may be used as rule-in or rule-out criteria for clinical decision-making. Typically, P2-bMRI phenotypes are based on the assessment of multiple criteria in concert as in this example: here, a machine learning algorithm was used to identify phenotypes predictive of nodal-positive or nodal-negative stage (N+, N-). Semantic imaging criteria of the index lesion were used to predict nodal stage (for details, please see reference [42]). Classification results are presented as an intuitive and easy to follow decision tree. Accordingly, the “nodal-negative P2-bMRI phenotype” is characterised by a smooth lesion without oedema and without skin thickening. The positive likelihood of N+ is 0% for this P2-bMRI phenotype. Similar results can be achieved with other predictive/prognostic MRI methods, including artificial intelligence, each of them providing intrinsic advantages and disadvantages

An example of the use of P2-bMRI for phenotyping using artificial intelligence approach has been proposed by Dietzel et al. [101] (Fig. 8). Authors aimed to predict axillary lymph node metastases based on semantic MRI parameters of the index cancer. To support clinical application, prediction was based on a minimal number of MRI descriptors, and machine learning methods were used for this purpose [2, 99]. As expected, a single MRI parameter (skin thickening) was already able to predict the risk of axillary lymph node metastases (Fig. 8, Table 1). However, a reliable rule-out criterion (*i.e.*, “no lymph node metastases”) could only be reached when three parameters were combined (Fig. 8, risk of nodal metastasis 0/56 = 0%).

### The future

To translate P2-bMRI into P4 breast cancer care, three major challenges should be overcome.

First of all, methodological development of P2-bMRI needs to be refined. It may be achieved at the level of MRI data analysis. Radiomics of individual lesions (and machine learning applied to lesion radiomic data) as well as the use of convolutional neural networks applied to the whole image(s) may be regarded as most promising tools here [15, 19, 69, 102]. Yet, there is still considerable potential for improvement even only based on semantic criteria. Future development of P2-bMRI may also be achieved at the level of data acquisition, and magnetic resonance spectroscopy may be particularly promising here [65, 68].

However, methodological development by itself is not sufficient to translate P2-bMRI into clinical practice. Empiric evidence on P2-bMRI is generally derived from small, monocentric, and retrospective studies. Clinical application will request validation of P2-bMRI in a real-world oncological setting before adopting the methods. This calls for dedicated interdisciplinary, large, multicentre studies, perhaps also randomised controlled trials.

Finally, the breast imaging community itself should try to be a strong promoter in the process of translating P2-bMRI into clinical practice. The prognostic potential of imaging biomarkers is not yet sufficiently recognised outside the field of radiology. Only when radiologists and nuclear medicine physicians dedicated to breast imaging will be successful in convincing key stakeholder such as patients, clinical colleagues, healthcare providers, and MRI vendors, P2-bMRI will start to be an integral part of routine breast cancer care.

## Abbreviations

ADC: Apparent diffusion coefficient
BPE: Background parenchymal enhancement
DCIS: Ductal carcinoma *in situ*
DWI: Diffusion-weighted imaging
HER2: Human epidermal growth factor receptor 2
MRI: Magnetic resonance imaging
P2-bMRI: Predictive and prognostic breast magnetic resonance imaging
P4: Personalised, predictive, preventive, and participatory
